# The role of IL-38 in intestinal diseases - its potential as a therapeutic target

**DOI:** 10.3389/fimmu.2022.1051787

**Published:** 2022-11-03

**Authors:** Qiang Wang, Linna Ma, Caiping An, Steven G. Wise, Shisan Bao

**Affiliations:** ^1^ Department of Anatomy, School of Basic Medicine, Gansu University of Chinese Medicine, Lanzhou, Gansu, China; ^2^ Department of Pathology, School of Basic Medicine, Gansu University of Chinese Medicine, Lanzhou, Gansu, China; ^3^ Department of Haematology and Nephropathy, Gansu Provincial Hospital, Lanzhou, Gansu, China; ^4^ School of Medical Sciences, Faculty of Medicine and Health, University of Sydney, Sydney, NSW, Australia

**Keywords:** IL-38, intestinal mucosa, immunity, inflammation, colorectal cancer

## Abstract

IL-38, an anti-inflammatory cytokine, is a key regulator of homeostasis in host immunity. Intestinal immunity plays a critical role in defence against pathogenic invasion, as it is the largest surface organ and the most common entry point for micro-organisms. Dysregulated IL-38 activity is observed in several autoimmune diseases including systemic lupus erythematosus and atherosclerosis. The protective role of IL-38 is well illustrated in experimental colitis models, showing significantly worse colitis in IL-38 deficient mice, compared to wildtype mice. Moreover, exogenous IL-38 has been shown to ameliorate experimental colitis. Surprisingly, upregulated IL-38 is detected in inflamed tissue from inflammatory bowel disease patients, consistent with increased circulating cytokine levels, demonstrating the complex nature of host immunity *in vivo*. However, colonic IL-38 is significantly reduced in malignant tissues from patients with colorectal cancer (CRC), compared to adjacent non-cancerous tissue. Additionally, IL-38 expression in CRC correlates with 5-year survival, tumour size and differentiation, suggesting IL-38 plays a protective role during the development of CRC. IL-38 is also an independent biomarker for the prognosis of CRC, offering useful information in the management of CRC. Taken together, these data demonstrate the role of IL-38 in the maintenance of normal intestinal mucosal homeostasis, but that dysregulation of IL-38 contributes to initiation of chronic inflammatory bowel disease (resulting from persistent local inflammation), and that IL-38 provides protection during the development of colorectal cancer. Such data provide useful information for the development of novel therapeutic targets in the management of intestinal diseases for more precise medicine.

## The physiological and pathological role of IL-38

IL-38 belongs to the IL-1 superfamily, because there is more than 40% homology between IL-38 and the IL-1 receptor (IL-1Ra) (1), and is also classified in the IL-36 subfamily, as IL-38 also shares the IL-36 receptor (IL-36R) pathway. Constitutive IL-38 expression is detected in a wide range of tissues including heart, lung, intestine and skin (2), as well as in the lymphoid organs, e.g. spleen and tonsils (3) and some leucocytes (macrophage and Langerhans cells) (4).

IL-38, an anti-inflammatory cytokine, plays an important role in maintaining homeostasis by balancing pro- vs anti-inflammatory responses in the microenvironment (5). IL-38 supresses host inflammatory responses *via* inhibiting the production of pro-inflammatory cytokines/chemokines, as demonstrated when exogenous IL-38 was found to supress pro-inflammatory mediators including IL-6, IL-1β, CCL5 and CXCL10 in a humanized allergic asthma NOD/SCID murine model *in vivo* (6). In addition, IL-38 decreases production of pro-inflammatory mediators (IL-17 and IL-22) from human mononuclear cells in response to microbial stimulation, synergistic with IL-36Ra (7) *via* NFKB pathway activation, and subsequently maintains the homeostasis of the microenvironment (8). IL-38 is also analogous to the IL-1ab/receptor agonist and IL-1R1, mediating anti-inflammatory activities. It has been further illustrated that IL-38 regulates innate and adaptive immunity (9) perhaps *via* controlling activation of macrophages. IL-38 also acts in an autocrine fashion by releasing IL-38 from apoptotic cells to limit inflammatory macrophage responses (10).

Dysregulation of IL-38 may initiate inflammation due to an imbalance of pro- vs anti-inflammation factors in the microenvironment, causing inflammatory diseases. Up-regulated IL-38 expression is detected in many autoimmune diseases, including in inflamed skin (2), the active inflamed tissues of inflammatory bowel disease (11), rheumatoid arthritis joints (12), psoriatic skin (5) and in drug-induced liver injury patients (13). These findings suggest that the increased IL-38 in these inflamed organs may be in response to these chronic focal stimuli, but that its presence is unable to maintain the inflammatory balance sufficiently. Interestingly, IL-38 is downregulated in psoriatic skin in response to stimulation by IL-36γ, IL-17 and IL-22n (all pro-inflammatory cytokines) (14). This observation suggests that IL-38 -counteracts the biological processes induced by pro-inflammatory cytokines in epithelial and endothelial cells to attenuate the severity of autoimmunity.

## Intestinal mucosal immunityand homeostasis

The intestinal mucosa has the largest surface of any organ in the body, estimated to be 170 m^2^ (15) and is the most common point of invasion for micro-organisms. Thus the intestinal mucosal surface is the most vulnerable towards both specific and non-specific (opportunistic) microbe challenges (16). Intestinal mucosal immunity, including cellular and humoral responses, provides a critical role in defence against microbial invasion, as well as in maintaining homeostasis to prevent autoimmunity (17).

The key role of intestinal humoral response has been demonstrated, with evidence that humoral mucosal specific and non-specific responses are compromised in IL-6 deficient mice (18), supported by evidence of compromised host defence against *pseudomonas aeruginosa* challenge in the mucosal epithelial surface on the cornea of IL-6 deficient mice (19, 20). Moreover, exogenous IL-6 boosts specific intestinal immunity (21). On the other hand, IFNγ plays a key role in intestinal cellular immunity against intracellular *Salmonella typhimurium* challenge *via* specific CD8^+^ cytotoxic T cells and CD4^+^ helper T cells (22), with deficiency of IFNγ compromising the activation of intestinal mucosal macrophages which are critical in killing engulfed *Salmonella typhimurium*, despite the substantially elevated specific and non-specific humoral responses in the *S typhimurium* challenged gut (22). Consistent with this finding, it has been demonstrated that the recruitment of activated macrophages plays a critical role in host cellular immunity, particularly macrophage mediated defence against *Salmonella typhimurium*, using GM-CSF deficient mice (23). *S typhimurium* can spread to the liver and spleen in the absence of GM-CSF, accompanied by a large number of infiltrating inactivated monocytes. In addition, chemokines such as CXCR-3, contribute to host defence in response to *S typhimurium* oral challenge, with deficiency of CXCR-3 leading to compromised intestinal mucosal immunity (24). Thus, host intestinal cellular and humoral immunity plays a critical protective role in defence against pathogenic invasion. These observations suggest that balance between pro- and anti-inflammatory mediators is critical in maintaining a disease-free environment on the intestinal surface.

## IL-38 in intestinal mucosal immunity

The therapeutic role of IL-38 has been demonstrated, with exogenous IL-38 ameliorating the severity of collagen induced arthritis (25) in a dose dependent manner. This is further supported by findings from IL-38 deficient mice, which have substantially more severe arthritis, compared to wildtype mice (12), accompanied by the increased presence of pro-inflammatory cytokines, including IL-1β and IL-6. However, there is also significantly higher IL-38 in the synovial fluid of rheumatoid arthritis patients (12), likely due to the hosts attempt to counteract the upregulated production of pro-inflammatory mediators in the chronically inflamed microenvironment (12). This is in line with the finding that circulating IL-38 is significantly upregulated in rheumatoid arthritis patients both at the protein and mRNA levels, compared to a non-rheumatoid arthritis cohort (26). The increased circulating IL-38, however, is inhibited in response to standard disease-modifying anti-rheumatic drug treatment (26), perhaps reflecting reduced host inflammatory status in response to effective management. We believe elevated IL-38 is due to the host response to chronic inflammation in the focal lesion, which in this condition is unsuccessful in suppression of the development of chronic inflammation in the affected joints. This is consistent with the observation that IL-38 reduces pro-inflammatory mediators, such as IL-8, IL-17A and IL-22 (7). However, the precise pathogenesis of IL-38 in rheumatoid arthritis remained to be definitively determined.

There is not yet published data to demonstrate the role of IL-38 in intestinal mucosal infection, but significantly upregulated circulating IL-38 from COVID-19 patients with severe lung viral infection of SARS-Cov2 has been reported and is of interest, since lung and gut are both mucosal associated lymphoid organs (27). The study from SARS-Cov2 infected mucosa of the lung invites speculation that increased IL-38 is trying to protect the target organ(s) from cytokine storm damage *via* suppressing the activities of pro-inflammatory cytokines (28). The observation of increased circulating IL-38 following lung viral infection of SARS-Cov2 virus from COVID-19 patients, is consistent with elevated serum IL-38 from chronic hepatitis B viral infected patients (29). Such data suggests that IL-38 could be a useful agent in intestinal mucosal immunity, particularly among these severe diseased patients from the salmonella outbreak (30). The precise underlying mechanism will be further investigated in humans and in animal model, as described (21, 23, 24).

Evidence for the protective role of IL-38 in intestinal mucosal immunity is demonstrated by substantially more severe clinical presentations (both systemic and local) and histopathological damage in intestinal mucosa from the IL-38 deficient mice, accompanied with more pro-inflammatory cytokines/chemokines (31) in response to dextran sulfate sodium (DSS) challenge, compared to wildtype mice. On the other hand, exogenous IL-38 ameliorates DSS induced colitis with substantially reduced systemic and local pro-inflammatory mediators (32). Such data strongly supports the critical protective role of IL-38, particularly in maintaining intestinal mucosal immunity *via* keeping homeostasis.

Human inflammatory bowel disease (IBD), including ulcerative colitis and Crohn’s disease, is characterised by chronic persistent gastrointestinal inflammation (33). Ulcerative colitis affects the superficial two layers of descending and sigmoid colon only, whereas Crohn’s disease can affect any part of gastrointestinal system (from mouth to anus), and all four layers of the intestinal wall (34). The clinical presentations include abdominal pain, diarrhea, rectal bleeding, and the severity is dependent on the remission and relapse of inflammatory bowel disease. The precise aetiology of IBD is complex and likely includes a combination of host genetical susceptibility, intestinal microenvironment, environmental triggers and the dysregulation of the intestinal immune system. However, there is substantial upregulation of IL-38 in the intestinal mucosa from IBD patients (11, 35) at both molecular and cellular levels, correlating well with the disease severity, although IL-38 is classified as anti-inflammatory cytokine. The discrepancy of the possible role of IL-38 in colitis between experimentally induced colitis in animal models (31, 32) and human IBD (11, 35) may have several causes. First, disease progression in experimentally induced colitis is only 1-3 weeks in animals with acute progression; whereas the course of human IBD is usually is over 10 years. Second, experimental colitis is induced with a chemical compound, which abruptly destroys the mucosal intergrade, resulting in an influx of gut flora and causing acute intestinal inflammation; whereas the cause(s) of human inflammatory bowel disease is very complicated, including environmental triggers, genetical susceptibility and local mucosal dysregulated immunity (33). Accordingly, we propose that the anti-inflammatory role of IL-38 is useful for the management of acute inflammation, but it becomes problematic in chronically susceptible individuals. It remains to be explored if upregulated colonic IL-38 result from high local inflammation, as the host tissue attempts to suppress the inflammatory response, but unsuccessfully. It might be the fact that the target(s) in the intestinal epithelial cells are not responded well to IL-38 at any level, which may also relate to in the downstream signalling pathway, e.g. IL-36R and downstream targets, in addition unknown environmental and/or unknown stimuli. Subsequently inflammation is persistent, trigging even higher inflammation.

Nevertheless, such finding suggests that IL-38 may be a target for potential therapeutic approach in the management of intestinal diseases, that is, exogenous IL-38 would be useful in treating acute and/or chronic intestinal infection (23, 24) and/or colorectal cancer patients (36), particularly among the immune-compromised patients, such as the elderly and/or HIV patients. This proposal is supported by the findings that compromised cellular (22) or humoral (18) immunity may contribute to reduced intestinal mucosal immunity and from experimental induced arthritis animal model data showing that exogenous IL-38 ameliorates the severity of the inflammation (4). It is uncertain if exogenous IL-38 should be applied for the management of chronic inflammation in inflammatory bowel patients, as colonic IL-38 is substantially up-regulated among these IBD patients (11, 35). IL-38 involves downstream pathway, IL-36R, and target cells, and these should also be considered further for therapeutic targeting. Finally, it should be kept in mind if the intestinal mucosal four layers remain or are at least partially present for the recovery or remission.

## IL-38 in colorectal cancer

There is constitutive production of IL-38 in the colonic mucosa (11, 35, 36), which is primarily distributed in the cytoplasm of intestinal epithelium. However, the production of colonic IL-38 is significantly decreased in tissue from colorectal cancer, compared to that of adjacent non-CRC colonic tissue (36). In addition, there is a correlation between the colonic IL-38 and differentiation of CRC, that is, the poorer differentiation, the lower IL-38 production, implying that IL-38 provides protection during the development of colorectal cancer, perhaps *via* inhibiting inflammation in the microenvironment (3, 5). This explanation is supported by other studies showing that chronic uncontrolled inflammation promotes malignancy in the colon, due to persistent damage of epithelial cells (37), consistent with the report of a close correlation between inflammatory bowel disease and the development of CRC (38). Moreover, down-regulated colonic IL-38 suppresses anti-inflammatory function and consequently promotes inflammation in the microenvironment, a phenomenon demonstrated in IL-38 gene knockout mice, showing substantially up-regulated pro-inflammatory mediators in the colon (31). The protective role of IL-38 is further supported by studies showing that IL-38 provides protection against gestational diabetes mellitus-induced inflammation within the placenta (39).

The unsatisfactory outcomes for CRC patients (poor prognosis and overall survival time) to date is mainly due to the progressed stage of disease upon diagnosis (40) such that the size of CRC is large and invasion is deep (41). These findings are consistent with others, showing that there is an inverse correlation between colonic IL-38 production and the size of CRC (36), in line with the poor survival rate. There is also an inverse correlation between colonic IL-38 expression and TNM classification among CRC patients, further supporting the protective role of IL-38 during the development of CRC (36).

From a clinical point of view, the diagnosis of CRC on the right side of the colon is often later with larger tumour size than left side of colon, due to the capacious nature of the proximal colon (42). In addition, colonic IL-38 is 50% less for right-side CRC, compared to that of the left side (36), which may be due to the difference of size and/or differentiation between right and left colon, or may be related to embryonic differentiation of right and left colons (43). From a molecular point of view, it has been reported that there is more frequent of microsatellite instability (MSI) mutation from right colon CRC, contributing to higher levels of inflammation within the tumour microenvironment, than the left side. Low levels of colonic IL-38 may be a complex reflection of this higher level of inflammation. However, the relationship between MSI, IL-38 expression and CRC differentiation should be further explored.

Although it is reported that age is involved in the development of CRC (44), there is no correlation of colonic IL-38 between young and old CRC patients (36). The explanation for this observation is that there is so far only a single centre study with a limited number of CRC patients, which compromise the outcomes for the statistical analysis. In addition, the patient inclusion cut-off is at 70 years, which may be due to the majority of the CRC patients from their study being within a relatively old group, close to 65 years (36). While the precise underlying mechanism of IL-38 involvement in the progression of CRC remains to be determined, the observation from CRC patients invites speculation that colonic IL-38 protects the host from focal chronic inflammation and mucosal damage by counteracting with pro-inflammatory cytokines to balance homeostasis (14). However, the critical role of colonic IL-38 may be compromised among susceptible cohorts (45), due to either reduced local IL-38 and/or interference with downstream pathway(s). Subsequently, chronic dysregulated inflammation could be induced in the intestinal mucosa (11, 35) leading to compromise of intestinal mucosal immunity (46). CRC is therefore eventually developed following long-term inflammatory stimulation (37, 47), which is consistent with reports showing a close correlation between chronic inflammation and the development of gastrointestinal cancer (48). This explanation is supported by the observed correlation between reduced circulating IL-31 and local mucosal IL-31 in gastric cancer (49), further supporting that both local and systemic host immunities contribute to the progression of malignancies among the susceptible individuals.

Surprisingly there is a controversial finding regarding local IL-38 production from malignancy in lung, showing an inverse correlation between IL-38 expression and differentiation of lung adenocarcinoma (50), in addition to a correlation between IL-38 and TNM (poor prognosis) in lung adenocarcinoma. The discrepancy between colonic and lung IL-38 in malignancy remains to be clarified. Although both intestine and lung are classified as mucosal organs with protection from mucosal associated lymphoid organs, there are significant differences between the gut and lung, for example, there is a major difference in the load of microbes between lung and gut (51). In addition, it has been demonstrated that intestinal flora contributes to the lung defence against pathogenic invasion (51).

## IL-38 and polarisation of tumour associate macrophages

Following the development of cancer there are large number of infiltrating leucocytes, including macrophages, neutrophils and lymphocytes (52), likely related to cancer surveillance mechanisms. However, these infiltrating leucocytes may not be sufficient to play this role in susceptible individuals, subsequently allowing escape from such surveillance and development of cancer. It is well documented that there are a considerable number of macrophages recruited in the tumour (termed tumour associated macrophages, TAMs). The roles of TAMs is debatable, that is whether they promote or inhibit growth of tumours (53), which may be related to the terminal differentiation of these macrophages. TAMs can be classified by their terminal differentiation into either classical activated M1 macrophages or alternatively activated M2 macrophages (54). It has been demonstrated that M1 TAMs display anti-tumour functions *via* direct free radical mediated cytotoxicity, releasing ROS and NO, and/or contribute to antibody-dependent cell-mediated cytotoxicity (ADCC). In contrast, M2 TAMs promote the occurrence and metastasis of tumour cells, perhaps *via* inhibiting the T cell-mediated anti-tumour immune response, promotion of tumour angiogenesis, leading to tumour progression (55). It has been reported that IL-38 promotes IL-35 mediated development of Treg cells, which may consequently enhance the development of cancer (56). However, it has also been demonstrated that IL-38 provides a protective role during the development of colorectal cancer (36). Such discrepancy in the described role of IL-38 in the development of cancer may likely relate to regulatory regulation of terminal differentiation of TAMs in the tumour microenvironments (55), dependant on the individual local and/or systemic responses. Therefore, it is important to explore the potential role of IL-38 in the management of intestinal diseases, perhaps *via* boosting M1, but inhibiting M2 macrophages.

In conclusion, colonic IL-38 plays a critical role in maintenance of local homeostasis in the intestinal mucosa. Compromised colonic production and/or function of IL-38, as well as its downstream pathway(s) may cause initiation of acute and/or chronic inflammation in the gut, with possible induction of malignancy if persistent inflammation remains. These insights highlight potential new molecular targets and pathways for clinical precision management of intestinal inflammation and/or malignancy.

## Author contributions

QW, LM, wrote the manuscript. CA conceptualized the manuscript. SW, SB revised the manuscript. All authors contributed to the article and approved the submitted version.

## Funding

Author SW is funded by a fellowship from the National Heart Foundation of Australia (105622).

## Conflict of interest

The authors declare that the research was conducted in the absence of any commercial or financial relationships that could be construed as a potential conflict of interest.

## Publisher’s note

All claims expressed in this article are solely those of the authors and do not necessarily represent those of their affiliated organizations, or those of the publisher, the editors and the reviewers. Any product that may be evaluated in this article, or claim that may be made by its manufacturer, is not guaranteed or endorsed by the publisher.
